# Supplementary material for: Extensive translation of small Open Reading Frames revealed by Poly-Ribo-Seq
Source: eLife. 2014 Aug 21;3:e03528. doi: 10.7554/eLife.03528 (PMC4359375; doi:10.7554/eLife.03528)
Supplement: Supplementary file 1. — (A) Summary of sequencing experiments. Number of reads; from each experiment, that are left after removal of rRNA and tRNA contaminants, that are unique matches and that map to CDS regions of the genome. (B) Summary of smORF embryo RNA-seq data. Number of translated smORFs expressed throughout embryonic stages of Drosophila melanogaster, according to RNAseq data (modENCODE). DOI: http://dx.doi.org/10.7554/eLife.03528.017 [file elife03528s001.docx]

Supplementary file 1

**A) Summary of sequencing experiments**

Number of reads; from each experiment, that are left after removal of rRNA and tRNA contaminants, that are unique matches and that map to CDS regions of the genome.

| Experiment | Raw reads | Reads that pass clipping and trimming | After removal of rRNA and tRNA | Tophat mapped reads | Unique match reads | Reads that map to ORFs |
| --- | --- | --- | --- | --- | --- | --- |
| Small polysomal  footprint | 99,973,197 | 93,838,021 | 6,250,297 (6.66%) | 5,498,415  (87.97%) | 3,748,512  (68.17%) | 2,870,975  (76.59%) |
| Large polysomal  footprint | 67,230,885 | 59,191,477 | 3,579,490 (6.05%) | 2,625,211  (73.34%) | 2,371,751  (90.34%) | 1,906,050  (80,36%) |
| Total cytoplasmic mRNA 1 | 3,737,677 | 3,296,829 | 2,249,793 (68.24%) | 2,006,335  (89.17%) | ND | 1,619,013  (80.7%) |
| Total cytoplasmic mRNA 2 | 8,698,975 | 4,374,205 | 2,911,316 (66.56%) | 2,559,302  (87.91%) | ND | 1,956,992  (76.47%) |
| Small  polysomal  footprint  extensive | 189,631,476 | 188,066,263 | 20,008,723 (10.64%) | 9,160,610  (45.78%) | 8,133,854  (88.79%) | 5,904,132  (72.59%) |
| -rRNA all polysomes  footprint | 14,092,100 | 12,759,694 | 4,144,111 (32.48%) | 3,190,896  (77.0%) | 2,972,003  (93.14%) | 2,355,257  (79.25%) |

**B) Summary of smORF embryo RNA-seq data**

Number of translated smORFs expressed throughout embryonic stages of *Drosophila melanogaster,* according to RNAseq data (FlyBase).

| Number of Embryo Stages | Number of smORFs (%) |
| --- | --- |
| 12 | 88 (41.7%) |
| 11 | 16 (7.6%) |
| 10 | 11 (5.2%) |
| 9 | 17 (8.1%) |
| 8 | 9 (4.3%) |
| 7 | 8 (3.8%) |
| 1-6 | 47 (22.3%) |
| 0 | 15 (7.1%) |
| **Total** | **211** |
| NA | 17 |
