# Supplementary material for: Extensive translation of small Open Reading Frames revealed by Poly-Ribo-Seq
Source: eLife. 2014 Aug 21;3:e03528. doi: 10.7554/eLife.03528 (PMC4359375; doi:10.7554/eLife.03528)
Supplement: Supplementary file 2. — Primers used for rRNA depletion. DOI: http://dx.doi.org/10.7554/eLife.03528.018 [file elife03528s002.docx]

SUPPLEMENTARY FILE 2

**Primers used for rRNA depletion**

| **Primer** | **Sequence (5' to 3')** |
| --- | --- |
| 2S Rev | biotin-TACAACCCTCAACCATATGTAGTCCAAGCA |
| 5S Fw | GCCAACGACCATACCACGCT |
| 5S Rev | biotin-AAAAAGTTGTGGACGAGGCC |
| 5.8S Fw | AACTCTAAGCGGTGGATCAC |
| 5.8S Rev | biotin-CAGCATGGACTGCGATATGCG |
| **Set 1** |  |
| 18S Fw A | ATTCTGGTTGATCCTGCCAG |
| 18S Rev A | biotin-CAAGAATTTCACCTCTCGCGT |
| 18S Fw B | GACCGTCGTAAGACTAACTT |
| 18S Rev B | biotin-TAATGATCCTTCCGCAGGTTC |
| 28S Fw A | TTATATACAACCTCAACTCAT |
| 28S Rev A | biotin-AAGTATAGTTCACCATCTTTC |
| 28S Fw B | GATCAGGTTGAAGTCAGGGG |
| 28S Rev B | biotin-CATGCTCTTCTAGCCCATCTA |
| 28S Fw C | ACATATACTGTTGTGTCGATA |
| 28S Rev C | biotin-AAATACATAAATGCATCGTTT |
| 28S Fw D | TTGATTTGAAAATTTGGTATA |
| 28S Rev D | biotin-TCGAATCATCAAGCAAAGGAT |
| **Set 2** |  |
| 18S Fw A | CCGAGGCCCTGTAATTGGAAT |
| 18S Rev A | biotin-ATATGAGTCCTGTATTGTTATTTT |
| 18S Fw B | ATTGTGTTTGAATGTGTTTATGTAAG |
| 18S Rev B | biotin-AAGCATTTTACTGCCAACATGAAT |
| 28S Fw A | ATATAAGGACATTGTAATCTATTAGC |
| 28S Rev A | biotin-GGAAAAAATGCACACTATTCTCAT |
| 28S Fw B | GCGCTTAAGTTGTATACCTATAC |
| 28S Rev B | biotin-CATCCATTTTAAGGGCTAGTTG |
| 28S Fw C | GCGGGTGTTGACACAATGTGA |
| 28S Rev C | biotin- TAGGGCCATCACAATGCTTTGT |
| 28S Fw D | CAAAACGTTGTTGCGACAGCA |
| 28S Rev D | biotin-TCATTAGTAGGGTAAAACTAACC |
|  |  |
|  | Set 1 was used to generate 1 Kb fragments and Set 1 and Set 2 were used in combination to generate 500 bp fragments |
|  |  |
